# The Origins of GPCRs

**DOI:** 10.3390/ijms27156723

**Published:** 2026-07-28

**Authors:** Vsevolod V. Gurevich

**Affiliations:** Department of Pharmacology, Vanderbilt University, Nashville, TN 37232, USA; vsevolod.gurevich@vanderbilt.edu

**Keywords:** rhodopsin, GPCR, evolution, prokaryotes, eukaryotes

## Abstract

G protein-coupled receptors (GPCRs) are present in all kingdoms of life. GPCRs are the largest family of signaling proteins in animals, including humans. Numerous lines of indirect evidence suggest that eukaryotic light-sensitive rhodopsins (usually called type II), as well as other structurally similar non-visual GPCRs, trace their origins from prokaryotic rhodopsins (type I), possibly via receptors in unicellular eukaryotes that have a similar transmembrane core, even though existing sequence analysis software cannot detect homology. Bacterial and archaeal rhodopsins share the same overall design with eukaryotic GPCRs: a flexible core consisting of seven transmembrane α-helices with an extracellular N-terminus, an intracellular C-terminus, and three loops on each side of the membrane. Both families include many hundreds of members and apparently have expanded for billions of years, which makes the results of direct comparisons of the sequence and structure of these groups of proteins inconclusive. The distribution of residues with aromatic and ionizable side chains in prokaryotic rhodopsins and eukaryotic GPCRs was found to be similar. Established similarities suggest that shared origins are possible but do not unambiguously prove this hypothesis.

## 1. Introduction

G protein-coupled receptors (GPCRs) are the largest and arguably the most structurally variable and functionally versatile family of signaling proteins in animals [1]. All GPCRs consist of a characteristic transmembrane core of seven closely packed α-helices with an extracellular N-terminus, an intracellular C-terminus, and three extra- and three intracellular loops. The sizes of the parts of these receptors located outside the membrane vary in different GPCR subtypes, ranging from just a few residues to large multi-domain elements consisting of hundreds of residues. The longest of these are the N-termini of adhesion GPCRs. The record-holder is aptly named “very large G protein-coupled receptor” with a molecular weight of ~700 kDa [2] (for comparison, bovine rhodopsin consists of 348 residues and has a molecular weight of ~38 kDa).

Humans express ~800 different GPCR subtypes, including >300 non-odorant receptors [3]. Most mammals express an even greater number of distinct GPCR subtypes: for example, African elephant has ~2000 odorant receptors [4]. Although currently there are drugs that bind only ~120–130 human receptor subtypes [5], GPCRs as a group are targeted by about a third of clinically used drugs [6].

While it does not have obvious practical implications, the issue of the evolutionary origin of eukaryotic GPCRs is hotly debated. There is virtually a universal agreement that eukaryotic GPCRs, including light receptors rhodopsins (type II), trace their origins to a common ancestor [7,8]. Accordingly, the evolutionary tree of the GPCR superfamily is usually depicted with one root [8]. This root is often suggested to be rhodopsin, with class A GPCRs (rhodopsin-like, ~700 subtypes in humans) and other GPCR classes having evolved from it [9]. However, some researchers, also on the basis of GPCR sequence analysis, classified known GPCRs into three clades, stating that we cannot answer the question whether these clades have a common ancestor [10].

There are conflicting views on the relationship between prokaryotic (type I) rhodopsins and eukaryotic GPCRs, including type II rhodopsins. One point of view, based on the absence of detectable sequence homology, is that structural similarities are the result of convergent evolution of proteins with the same function [11]. However, caution must be exercised: is was established that amino acid substitutions in proteins accumulate at a rate that is expected to change the sequence beyond recognition by existing software during 1.8–2.7 billion years of evolution after eukaryotes descended from prokaryotes [12], that less than 30% sequence identity is usually considered inconclusive [13], and that 3D protein structures demonstrate much greater conservation than amino acid sequence [14]. Therefore, the opposite view, that prokaryotic rhodopsins and eukaryotic GPCRs have a common ancestor, is often expressed. To consider the pros and cons of the idea of common evolutionary roots of pro- and eukaryotic proteins sharing the core of seven transmembrane α-helices rationally, we need to place the evidence regarding these groups of proteins into the context of the evolution of eukaryotic cells from prokaryotic ancestors.

## 2. How Did Eukaryotic Cells Emerge?

The nucleus of prokaryotic cells (bacteria, including cyanobacteria, and archaea) is not separated from the cytoplasm by a lipid membrane. Prokaryotic cells also do not have membrane-delimited internal organelles. In contrast, in eukaryotic cells all internal organelles are surrounded by membranes that separate them from the bulk of the cytoplasm: the nucleus, mitochondria, chloroplasts and vacuoles in plants, lysosomes, endosomes, the endoplasmic reticulum, the Golgi apparatus, etc. [12]. The dominant view is that eukaryotic cells emerged as a symbiosis of anaerobic archaea with aerobic eubacteria that eventually became our mitochondria. Recent discoveries show that based on many molecular features Asgard archaea are the closest clade to eukaryotes, which suggests that eukaryotic cells likely evolved from this group of archaea (reviewed in [15,16]). Mitochondria in eukaryotic cells have their own bacterial-like circular genome and parallel protein synthesis machinery [12,17]. Direct comparison of specific features of protein-making ribosomes in mitochondria and the cytoplasm of eukaryotic cells supports the view of the archaeal origin of the cell and the eubacteria origin of mitochondria. Mitochondrial ribosomes are smaller and faster than ribosomes in the cytoplasm, clearly resembling those found in eubacteria. Having the same function, pro- and eukaryotic ribosomes share many features, but differ in several aspects [18]. Striking similarities between the functional cycle of cytoplasmic ribosomes in eukaryotic cells and archaeal ribosomes have been reported [19,20,21], further supporting the idea that eukaryotic cells evolved from archaea. Chloroplasts in plants apparently descended from endosymbiotic cyanobacteria: they also have a separate circular genome and their own bacterial-like ribosomes (recently reviewed in [22]).

Prokaryotic rhodopsins and eukaryotic GPCRs, including light-sensing type II rhodopsins, have seven transmembrane α-helices. Conceivably, integral membrane proteins in the plasma membrane of eukaryotes could have evolved from the proteins that were present in the plasma membrane of the host archaeal cells, from the membrane proteins of endosymbiotic bacteria and cyanobacteria, or from membrane proteins of various prokaryotic species acquired via horizontal gene transfer (see Section 5). Sequence analysis, however sophisticated, cannot distinguish between these possibilities.

## 3. Rhodopsins: Evolution Is Not in Doubt, but Was It Convergent or Divergent?

Existing bacterial and archaeal species express numerous rhodopsins performing a wide variety of light-driven functions. These include using the energy of light to pump protons and other ions across the membrane into or out of the cell, as well as intracellular signaling mediating light detection [23,24]. Structurally prokaryotic rhodopsins are quite diverse, as could be expected of the members of an ancient protein family, but they are usually considered divergent descendants of a single ancestral protein [25,26,27]. It has been shown that relatively minor sequence modifications of bacterial rhodopsins result in dramatic changes in their functions, including the direction of proton transport [28]. The common feature of type I rhodopsins is that their functions are always controlled by the absorption of light of a particular wavelength. The presence of seven transmembrane α-helices was demonstrated in bacteriorhodopsin from *Halobacterium halobium* almost 50 years ago [29], even before the amino acid sequence of this protein was determined [30]. The sequence of bovine rhodopsin revealed the same number of transmembrane helices and a tantalizingly similar distribution of aromatic residues, tryptophanes, phenylalanines, and tyrosines, in them [31]. Retinal, which is the actual light-absorbing moiety, is covalently attached via a Schiff base with an amino group of the lysine residue located in the seventh transmembrane helix in both types of rhodopsins [7]. However, existing sequence analysis software cannot detect reliable homology between prokaryotic (type I) and eukaryotic (type II) rhodopsins and other GPCRs. This led to the idea that the similarities between the two rhodopsin families are the result of convergent evolution [11].

**Figure 1:**
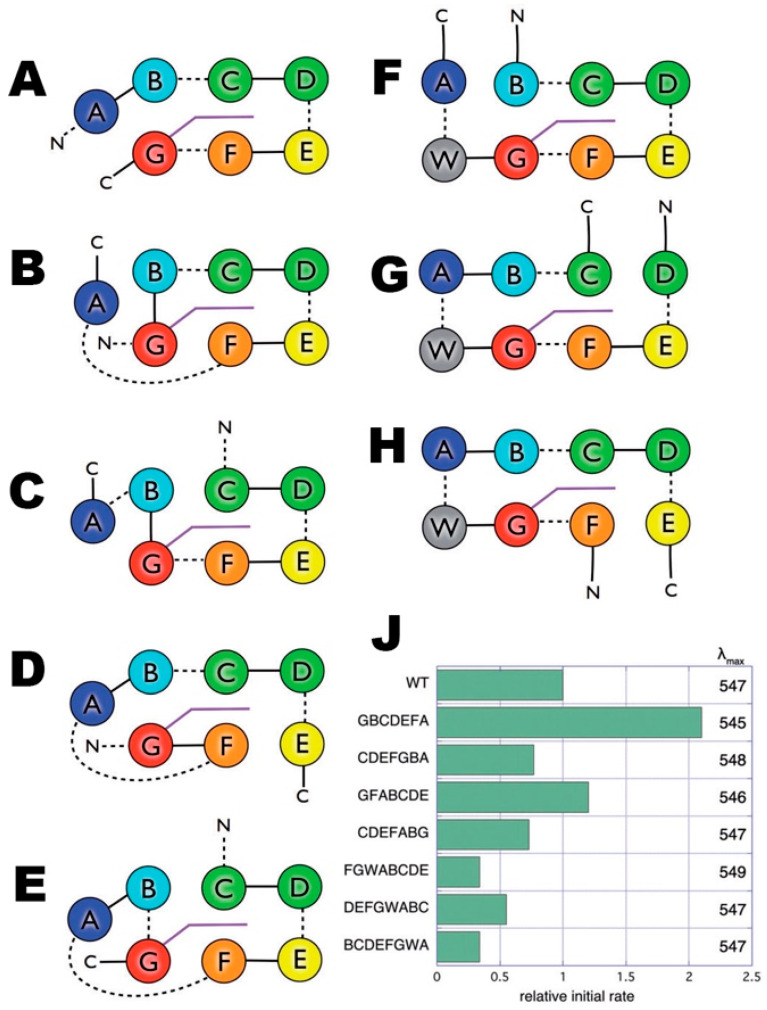
Bacteriorhodopsin transmembrane helix permutation constructs are functional. (**A**). Helix arrangement in wild-type bacteriorhodopsin. (**B**–**H**). Constructs where the transmembrane helices of wild-type bacteriorhodopsin were connected in different orders (as shown). The helices are labeled (A–G) according to their position in the wild-type bacteriorhodopsin and shown in the same color; an additional eighth helix introduced in several constructs is colored gray and labeled W. (**J**). Proton pumping activity and the wavelength of maximum absorption (λ_max_) of wild-type bacteriorhodopsin and permutation constructs. The figure elements are from [32], used by permission. The figure was assembled and labeled in Adobe Photoshop 2025 (Adobe, San Jose, CA, USA).

This idea implies that the order of the helices in the protein, as well as the position of the lysine residue for retinal attachment, are required for function. These implications were tested experimentally in bacteriorhodopsin [32] and bovine rhodopsin [33]. It turned out that the rearrangement of the sequence of helices within the primary structure of bacteriorhodopsin and even the introduction of an additional eighth helix yield fully functional light-driven proton pumps, two of which were found to be functionally superior to the original wild-type bacteriorhodopsin [32] (Figure 1). Moreover, reshuffling of transmembrane helices minimally changed the absorption maximum: by no more than 2 nm from the original 547 nm in wild-type bacteriorhodopsin (Figure 1). In these “scrambled” bacteriorhodopsins the retinal was attached via a Schiff base with the lysine in the same helix (which obviously was not seventh in these constructs). However, changing the position of retinal-binding lysine to the two other helices in bacteriorhodopsin [32], as well as to helix 2 or extracellular loop connecting helices 4 and 5 in bovine rhodopsin [33] also yielded functional light receptors. Modified versions of bovine rhodopsin with alternative retinal attachment sites were shown to be capable of activating cognate G protein rod transducin upon light exposure [33]. The efficiency of light-dependent transducin activation by the mutant combining Lys296Gly (that eliminates the original retinal attachment site) with Gly90Lys creating an alternative retinal attachment site in the second transmembrane helix approaches that of wild-type rhodopsin [33]. Moreover, rhodopsin with retinal attached to the lysines engineered in alternative positions demonstrated the same negligible constitutive (light-independent) activity towards transducin as the wild-type protein with retinal attached to Lys296 in the seventh helix [33]. Collectively, these results are inconsistent with the idea that convergent evolution recreated similar structures because of the same functional requirements. Thus, there must be other reasons(s) for structural similarities between pro- and eukaryotic rhodopsins.

Available data suggest that evolution has already taken advantage of the fact that rhodopsin remains efficient as a light receptor when retinal-binding lysine is localized in a different place. In UV-sensitive opsins from several insects the retinal is attached to the lysine in the second transmembrane helix [34], as in Gly90Lys bovine rhodopsin mutant [33]. Mammals also have UV-sensitive photopigments called neuropsins, which have a lysine residue in a nearby position homologous to Phe91 of bovine rhodopsin [35]. One of the neuropsins, OPN5, was shown to be a bistable (i.e., converted by light of different wavelengths between the two states, like invertebrate rhodopsins) UV-sensitive photopigment [36]. However, mammalian neuropsins, as well as UV-sensitive insect opsins, also have a lysine in the position homologous to Lys296 in bovine rhodopsin, and the actual site of retinal attachment in neuropsins has not been established [37]. From the evolutionary standpoint, the loss of the original lysine would yield a non-functional protein, so that to preserve light sensitivity an alternative retinal attachment point must emerge before the conventional one disappears. Although logical, this is a conjecture.

Based on the inferences of probable ancestral states of individual pro- and eukaryotic rhodopsins, it was concluded that both groups evolved from a common ancestor [13]. However, sequence homology of reconstructed probable ancestral proteins did not reach 30%, the level customarily considered to prove true homology [13], i.e., shared origin.

## 4. Light-Induced Signaling of Rhodopsins

Naturally, for a rhodopsin to serve as a signaling molecule it must either interact in its light-activated state with intracellular signal-transducing proteins [38] or contain on the cytoplasmic side an effector responding to the conformation of the light-sensitive part within the same polypeptide chain [39]. In fact, evolution used both mechanisms. Light sensitivity of flagellated swimming zoospores of the fungus *Blastocladiella emersonii* was shown to be mediated by a protein where a type I (bacterial-like) rhodopsin domain is fused to the guanylate cyclase, which is activated by green light captured by the rhodopsin moiety [39]. This arrangement ensures phototaxis of zoospores towards green light. Halophilic archaea (prokaryotes) have two sensory rhodopsins, unimaginatively named SRI and SRII (SR stands for sensory rhodopsin), that upon photoactivation interact with membrane-imbedded (containing two transmembrane helices) signal transducers HtrI and HtrII, respectively. The large cytoplasmic domain of these transducers binds histidine kinase CheA, which controls cytoplasmic flagellar motor switch regulator CheY (reviewed in [40]). SRI activated by orange light inhibits the activity of CheA, inducing an attractant response by preventing motor switching that changes the direction of swimming, whereas blue light has the opposite effect acting via SRII [40]. Upon photon capture vertebrate rhodopsins signal by activating a soluble transducer, heterotrimeric G protein called transducin (the name reflects its function); the GTP-liganded α-subunit of activated transducin activates cGMP phosphodiesterase; this reduces intracellular concentration of cGMP, which leads to the closure of cGMP-gated sodium/calcium channels that results in hyperpolarization of photoreceptor cells (recently reviewed in [41]). Invertebrate rhodopsins are also GPCRs: they activate Gq-like heterotrimeric G proteins, which in turn activate phospholipase C with consequent opening of transient receptor potential channels (reviewed in [42]).

Thus, both pro- and eukaryotic rhodopsins mediating light signaling were shown to use transducers encoded by different genes, so the mechanism of signal transduction does not shed light on their evolutionary origins.

## 5. Type I Rhodopsins in Eukaryotes

The evolution of type II rhodopsins from type I could occur only if eukaryotes expressed type I rhodopsins in their plasma membrane. There are two possible sources: type I rhodopsins could have been inherited from archaeal species that ultimately gave rise to eukaryotes, or they could have been added by horizontal (also called lateral) gene transfer (HGT) from prokaryotic species expressing these proteins. HGT is a fairly common phenomenon in all branches of life, including eukaryotes from yeast to humans [43,44]. In fact, cross-kingdom HGT was proposed to be one of the main drivers of plant evolution [45] with a profound impact on adaptation and functional diversification [46]. Sequence analysis of type I rhodopsin-like genes suggests that prokaryotic rhodopsins are expressed in a variety of eukaryotic organisms [25]. It was estimated that from 2 to 4% [45] to 36–49% [47] of plant genes are of prokaryotic origin. Several mechanisms of HGT were proposed. Intuitively, the most obvious is the acquisition of a gene from an endosymbiont. A significant number of genes appears to have been transferred from the mitochondrial to the eukaryotic genome localized in the nucleus [48,49]. In-depth analysis showed that as much as 18% of the genome of a small flowering plant *Arabidopsis thaliana* (belonging to the *Brassicaceae* family, to which various species of cabbage and mustard also belong) likely originated by gene transfer from the cyanobacterial ancestor of its chloroplasts [50]. A more recent analysis of the genomes of *Arabidopsis thaliana* and *Oryza sativa* (Asian cultivated rice) showed that the total fraction of protein-coding genes of prokaryotic origin in these plants is even greater, 36.5% and 49.3%, respectively [47]. Another possible mechanism of HGT is the acquisition of genes from food. HGT from ingested organisms to phagotrophic species was shown to be widespread [43]. DNA-containing extracellular membrane vesicles are yet another way of gene transfer between prokaryotic and eukaryotic species [51,52]. Pieces of genomic DNA can also be packaged into viral particles instead of the viral genome and transferred to cells targeted by these particles [53]. Viruses routinely exchange genetic material with their hosts [54]. Thus, another conceivable mechanism is that a virus inserting into the genome leaves while taking neighboring genes with it. Via this mechanism a genome-inserting virus that is shared by two or more species can mediate gene transfer between them. There are many additional known mechanisms of HGT, such as transposable elements (transposons) [55], plasmids [56], as well as very likely others that are still unknown [43]. Of course, the occurrence of HGT events that happened hundreds of millions of years ago cannot be proved beyond a reasonable doubt today.

Prokaryotic rhodopsins utilize the conversion of all-trans-retinal to 13-cis-, whereas type II rhodopsins in the dark contain 11-cis-retinal that is converted by the absorbed photon to the all-trans form [57]. Quantum yields in all rhodopsins exceed 60%, whereas quantum yields of free retinal conversion range from 10% to 30% [57]. This difference indicates the importance of the protein environment for increased quantum yields, as well as for the documented faster photoconversion of protein-attached retinal [57]. Interestingly, one of the rhodopsins in halophilic archaea *Haloquadratum walsbyi* uses 11-cis-retinal as a chromophore [58]. Thus, the switch of the type of retinal isomer used could have occurred in rhodopsins of archaeal ancestors before they evolved into eukaryotic receptors. The expression of type I (prokaryotic) rhodopsins, either inherited from archaea or added by HGT, in the plasma membrane was documented in several eukaryotic taxa, such as fungi [39], algae [59], dinoflagellates [60], stramenopiles, haptophytes, and cryptophytes [61].

To summarize, existing evidence demonstrates that many modern eukaryotes express type I rhodopsins, which, regardless of their provenance, could have served as the starting material for the evolution of type II rhodopsins and other eukaryotic GPCRs. Considering the length of eukaryotic evolution (eukaryotic cells are believed to have emerged 1.8–2.7 billion years ago [12]) and the remarkable tolerance of the seven transmembrane helix design to sequence perturbations [32,33,37], accumulated mutations could have changed the amino acid sequence so much that existing software is incapable of detecting homology [7].

Thus, many modern eukaryotic species express type I rhodopsins of prokaryotic origin. This suggests the possibility that eukaryotic GPCRs with the same topology could have evolved from prokaryotic ancestors. However, this does not prove that they did.

Interestingly, it appears that gene flow in the opposite direction, from eukaryotes to prokaryotes, also exists. Unusual rhodopsins with reversed topology (intracellular N-terminus and extracellular C-terminus), termed heliorhodopsins or type 3 rhodopsins, were discovered in eukaryotes and prokaryotes [25]. These proteins demonstrate sequence homology with prokaryotic type I rhodopsins, particularly those expressed in dinoflagellates, which are eukaryotes, and genomic areas surrounding heliorhodopsin genes in prokaryotes contained genes of eukaryotic origins [25].

## 6. GPCR Superfamily

GPCRs are signaling proteins expressed in all eukaryotic kingdoms: plants [62], yeast [63], other fungi [64], the slime mold *Dictyostelium discoïdeum* [65,66], protozoa and early metazoa [67,68]. In animals these receptors are particularly numerous, with a large fraction of genes encoding different GPCR subtypes [1]. Because of the abundance of chemoreceptors (equivalent to odorant receptors) necessary for its specific lifestyle, GPCR genes were reported to constitute ~5% of all protein-coding genes in the roundworm *Caenorhabditis elegans* [69]. The most recent gap-free sequence of *C. elegans* genome identified ~20,000 protein-coding genes [70]. The estimated number of chemoreceptor genes (1300–1700) [71] suggests that this fraction is even greater.

Structurally the GPCR superfamily is extremely diverse. Human GPCRs were divided into five families [3]. Members of four of these, rhodopsin-like, adhesion, glutamate, and frizzled, were identified in fungi [72], suggesting that diversification within this receptor family occurred before the separation of fungal, plant, and metazoan lineages in eukaryotic evolution. Universal residue numbering in the transmembrane helices of rhodopsin-like GPCRs, “anchored” on the most conserved residues in each helix, was proposed [73]. This nomenclature was extended to other classes of GPCRs [74] and is now widely used in the field. Curiously, even though this nomenclature was proposed for the helices crossing the hydrophobic lipid bilayer, three of the “anchoring” most conserved residues are hydrophilic (Asn, Asp, and Arg in helices 1, 2, and 3, respectively). Three more are prolines that disrupt α-helical structure, introducing kinks (in helices 4, 5, and 6). Only in helix 4 a semi-hydrophobic tryptophan (which is capable of H-bonding, in contrast to truly hydrophobic residues like phenyalanine, leucine, isoleucine, valine, etc.) was suggested to serve as an “anchor” [73]. As could be expected in the protein family that evolved for more than a billion years, primary structures even within a single subfamily of rhodopsin-like GPCRs are so divergent that their phylogeny cannot be established solely by methods based on sequence analysis of the whole protein [75]. However, the alignment of transmembrane helices across human GPCRs belonging to different subfamilies appears to be possible [76]. This required focusing on the chemical nature of the side chains, rather than on the amino acid identity [76].

Different hypotheses regarding the evolution of eukaryotic GPCRs from prokaryotic rhodopsins were proposed. On the basis of the documented binding of sodium ions to many GPCRs, it was suggested that eukaryotic receptors evolved from prokaryotic sodium-pumping rhodopsins [77]. Another analysis suggested that glutamate receptors (class C) are closer to prokaryotic rhodopsins than other GPCRs [78]. The authors concluded that transmembrane core evolved from bacteriorhodopsin, while the extracellular Venus flytrap domain (which all class C GPCRs have) evolved from structurally similar bacterial periplasmic binding protein [78]. Although vertebrate rhodopsin is widely considered a prototypical GPCR, this reflects the history of the discovery of the molecular mechanism of receptor signaling but does not necessarily reflect phylogeny. Based on the finding that retinal-binding lysine in the seventh transmembrane helix of metazoan rhodopsins is shifted by three residues relative to prokaryotic proteins (where an equivalent position is occupied by an aspartic acid), it was suggested that in evolution prokaryotic rhodopsin lost this lysine, whereupon the resulting protein diversified into various receptors, with one of the branches that gave rise to metazoan rhodopsins acquiring a lysine capable of retinal binding, thereby reacquiring light sensitivity [13]. This hypothesis implies that retinal attachment to the seventh transmembrane helix is functionally important, which is not supported by experimental evidence [32,33,37]. Structural analysis suggested that within the membrane-spanning core prokaryotic rhodopsins and eukaryotic GPCRs share a network of buried ionizable side chains [79]. This network is used by prokaryotic rhodopsins to pump ions across the membrane, whereas in eukaryotic GPCRs, both responding to light and diffusible ligands, this network is characteristic of activated conformations [79].

Although today it belongs to the category “everybody knows that,” the concept of GPCRs, i.e., of the existence of a large family of rhodopsin-like receptors using similar signaling mechanisms, emerged only in 1986, when determined sequence of β_2_-adrenergic receptor revealed its homology with rhodopsin [80]. This finding had profound conceptual importance because it suggested that molecular mechanisms underlying receptor signaling and its regulation that were earlier described in rod photoreceptors (recently reviewed in [41]) likely apply to other homologous receptors. This idea was confirmed by numerous subsequent studies (reviewed in [81,82,83]). Although odorant GPCRs (incidentally, these belong to class A, i.e., rhodopsin-like receptors) are the most numerous groups in vertebrates and invertebrates, hundreds of non-odorant subtypes respond to a wide variety of stimuli, from photons of light of different wavelengths (rhodopsin-like photopigments) to hormones, neurotransmitters, peptides, proteins, extracellular calcium, protease activity, adhesion to other cells or extracellular matrix, etc.

Thousands of metazoan GPCRs have been sequenced, including >800 human GPCRs [3,76]. The first classifications included all proteins with seven transmembrane α-helices, dividing them into families (clans) A, B, C, D, E, and F [84,85]. Humans do not have receptors belonging to clans D, E, and F (which include fungal pheromone receptors, cAMP receptors, and archaebacterial opsins, respectively). Human GPCRs were classified into five families: glutamate, rhodopsin-like, adhesion, frizzled, secretin (the GRAFS system) [3]. This classification was based on the analysis of sequences of transmembrane regions, ignoring extra- and intracellular receptor elements, which are the most variable in the family. Even focusing exclusively on transmembrane regions left >20 human receptors with seven transmembrane α-helices outside the five families [3]. The authors showed that based on pure sequence homology the relatedness of the receptors within each family has strong bootstrap support, while the common ancestry of all five families does not [3]. Manual alignment of transmembrane helices revealed signature sequence motifs within each family, many of which are shared by two or more families. Based on this, the authors hypothesized that all GPCRs trace their origin to a common ancestor [3]. Since sequence alignment alone did not unambiguously reveal the phylogeny within human GPCRs, in the newer analysis the information on sequences of the transmembrane regions was supplemented with emerging structural information [76]. This analysis revealed that GPCRs of different families share highly conserved 3D fold of their transmembrane helices, as well as numerous conserved contact points between helices [76]. Importantly, these contact points were identified by considering only the chemical nature of the side chains involved, ignoring the amino acid identity. The authors noted that many disease-causing mutations in GPCRs involve the residues identified as helix-to-helix contact points [76]. A remarkably conserved cysteine in all families (Cys3.25 in Ballesteros-Weinstein nomenclature [73]) forms a disulfide bond with a cysteine in extracellular loop 2 [76], which appears to be important for GPCR folding and stability. Phylogenetic tree constructed on the basis of this analysis (in A-F system based on the hypothesis of common origin) suggested that class C branched off first, then class B and adhesion receptors, then class F, with the rest of the tree consisting of class A GPCRs [76]. This is consistent with the estimates suggesting that class C and cAMP receptors are the oldest (older than 1.4 billion years), followed by classes B and F (~1.275 billion years), while class A (rhodopsin-like) is the youngest (~1.1 billion years) [72]. Note that the analyses in the studies hypothesizing common ancestry of all proteins containing seven transmembrane helices were focused on these helices, deliberately excluding extra- and intracellular GPCR elements.

The characteristic seven transmembrane α-helix design of prokaryotic rhodopsins and eukaryotic GPCRs turned out to be a huge evolutionary success [69], likely due to the remarkable flexibility of the GPCR transmembrane core, which was experimentally demonstrated by biophysical methods in case of rhodopsin [86], β_2_-adrenergic [87] and M2 muscarinic [88] receptors. GPCR activation results in the opening of an inter-helical cavity on the cytoplasmic side. This structural rearrangement was discovered in rhodopsin [89] and later shown to be shared by non-visual GPCRs (reviewed in [90]). All known cytoplasmic proteins that preferentially bind activated GPCRs, G proteins [91], GPCR kinases [92], and arrestins [93] were shown to dock to this cavity, in addition to the interactions with other elements on the cytoplasmic side of the receptors. Apparently, the opening of this cavity serves as a sign of receptor activation for the binding partners. It should be noted that prokaryotic rhodopsins, regardless of their function, respond to light, whereas eukaryotic GPCRs evolved to respond to diverse stimuli, from photons, ions, and small molecules to the binding of large proteins or even site-specific proteolytic cleavage of their N-termini.

Remarkable versatility of GPCRs, critical role of GPCR subtypes in virtually every physiological function, as well as convenient for targeting with small molecules (druggable) extracellular ligand binding pocket in these receptors explain why a huge proportion of clinically used drugs targets various members of GPCR superfamily [6]. Their biological and clinical importance explains a broad interest of the scientific community in the evolutionary origins of GPCRs.

## 7. Conclusions

Reconstructing the past is as hard as predicting the future: too many uncertainties are involved. There are structural similarities between the family of light-sensitive prokaryotic rhodopsins and the superfamily of eukaryotic GPCRs that include metazoan rhodopsins: shared seven transmembrane α-helix design of the protein core and similar distribution of residues with aromatic and ionizable side chains within these helices. These similarities are explained by convergent evolution by some and by common ancestry of these two protein families by others. Even though numerous species in several eukaryotic lineages express type I rhodopsins of prokaryotic origin, it is virtually impossible to prove either view beyond reasonable doubt: both groups of proteins with the core of seven transmembrane α-helices are huge and diverse, having evolved for billions of years. However, while undoubtedly possible, it does not appear probable that the evolution “invented the wheel” several times. In most cases upon “invention” the evolution uses and reuses successful protein designs for different purposes, as evidenced by the documented presence of shared domains in numerous functionally unrelated protein families. This consideration makes the idea that prokaryotic type I rhodopsins and eukaryotic GPCRs have common evolutionary roots plausible. Admittedly, existing evidence does not unambiguously prove this hypothesis. In fact, it is hard to even conceive the evidence that can be considered definitive proof. More sophisticated analysis of sequence similarities, including comparison of probable ancestral states, is unlikely to become more conclusive when applied to large and evolutionarily old protein families. Broader sampling of receptors in older eukaryotic lineages is unlikely to help, as all proteins in modern organisms are removed from their ancestors by the same amount of time. Structural similarities also do not prove ancestry, as these can be convincingly explained by convergent evolution. Thus, the hypotheses of divergent and convergent evolution will coexist in the foreseeable future.

## Data Availability

No new data were created or analyzed in this study. Data sharing is not applicable to this article.
